# 

**Published:** 1916-04-01

**Authors:** 


### CONTENTS.

PAGE

**The Medical Profession and the Army...** ... 1

**Orthodoxies and Heresies** ... ... ... ... 2

**Hospital and Institutional News** ... ... ... 3

The Royal Colleges and the Central Medical War Council—Mr. Hughes and Wounded Australians A Curious Point in Law—An Architect's Munificence—Scotland's First Open-Air Hospital—Dental Surgery at Guy's Hospital—A Leaflet on Measles—Another Poor-Law Institution as a Military Hospital—Mr. Baker's Reward at Hertford—The Prosperity of Industrial Towns—Economy at Bideford—The Children of Dundee—The Eyes of Munition Workers—A Chemist's Tragic Mistake—Juvenile Delinquency—Dispensers and Military Service—Hospitals and Public Libraries—This Week's Drug Market.

**Tuberculosis in Wales** ... ... ... ... ... 7

Activities of the National Memorial Association.

**The Chemical Treatment of Tuberculosis** ... 8

**Military Medical Notes** ... ... ... ... 9

**Education and Infant Welfare** ... ... ... 10

The Views of the Board of Education on Mothercraft.

**Textile Supplies for Hospitals** ... ... ... 13

VIII. Sheets and Quilts.

PAGE

**The Institutional Library** ... ... ... ... 11

Construction, Equipment, and Management of a General Hospital—A Manual for Nurses—The Health of the Skin—The Primary Lung Focus of Tuberculosis in Children—Physiology for Nurses — *Post-Mortem* Methods — Maternity. Letters from Working Women.

**The Matrons' and Sisters' Department...** ... 15

"Poking Fun at the Matrons."
The College of Nursing and State Registration.
Round the Hospitals.
King George on his own—The Buckingham Palace Party—Sir F. Milner's Visits—The Editor and his Readers—More about that Album—How to Help and Bleed your Chief.

**Parliament and the Profession** ... ... ... 18

**The Editor's Letter-Box** ... ... ... ... 19

Linen Leagues for Hospitals.

**Charing Cross Hospital** ... ... ... ... 19

**Annual Meetings of Special Interest** ... ... 19

**Passing Events and Topics** ... ... ... ... 20

**Hospital Results in 1915** ... ... ... ... 20

**The Institutional Worker** ... ... (Suppl.) 1

How We Nearly Doubled our Income in Two Years.
Question for April.
Inquiries and Answers.

